# Neuromuscular Mechanisms of Motor Adaptation to Repeated Treadmill-Slip Perturbations During Stance in Healthy Young Adults

**DOI:** 10.1109/TNSRE.2024.3485580

**Published:** 2024-11-28

**Authors:** Shuaijie Wang, Rudri Purohit, Tamaya Van Criekinge, Tanvi Bhatt

**Affiliations:** Department of Physical Therapy, University of Illinois at Chicago, Chicago, IL 60612 USA; Department of Physical Therapy and the Ph.D. Program in Rehabilitation Sciences, College of Applied Health Sciences, University of Illinois at Chicago, Chicago, IL 60612 USA; Department of Rehabilitation Sciences, KU Leuven, 8200 Brugge, Belgium; Department of Physical Therapy, University of Illinois at Chicago, Chicago, IL 60612 USA

**Keywords:** EMG, muscle synergy, muscle coordination, slip adaptation, treadmill slip perturbation

## Abstract

Treadmill-based repeated perturbation training (PBT) induces motor adaptation in reactive balance responses, thus lowering the risk of slip-induced falls. However, little evidence exists regarding intervention-induced changes in neuromuscular control underlying motor adaptation. Examining neuromuscular changes could be an important step in identifying key elements of adaptation and evaluating treadmill training protocols for fall prevention. Moreover, identifying the muscle synergies contributing to motor adaptation in young adults could lay the groundwork for comparison with high fall-risk populations. Thus, we aimed to investigate neuromuscular changes in reactive balance responses during stance slip-PBT. Lower limb electromyography (EMG) signals (4/leg) were recorded during ten repeated forward stance (slip-like) perturbations in twenty-six young adults. Muscle synergies were compared between early-training (slips 1–2) and late-training (slips 9–10) stages. Results showed that 5 different modes of synergies (named on dominant muscles: W_TA_, W_S_VLAT_, W_R_GAS_, W_R_VLAT_, and W_S_GAS_) were recruited in both stages. 3 out of 5 synergies (W_TA_, W_R_VLAT_, and W_S_GAS_) showed a high similarity (r > 0.97) in structure and activation between stages, whereas W_R_GAS_ and W_S_VLAT_ showed a lower similarity (r < 0.83) between the two stages, and the area of activation in W_TA_, the peak value of activation in W_R_VLAT_ and the activation onset in W_R_GAS_ showed a reduction from early- to late-training stage (p < 0.05). These results suggest that a block of stance slip-PBT resulted in modest changes in muscle synergies in young adults, which might explain the smaller changes seen in biomechanical variables. Future studies should examine neuromuscular changes in people at high risk of falls.

## I. INTRODUCTION

About one-third of the older adults experience at least one injurious fall each year [1], and the risk is more significant in neurologically impaired populations, such as those with stroke and multiple sclerosis [2], [3]. Fall-related injuries can have serious socioeconomic impacts on individuals, including loss of independence and increased caregiver burden, as well as significant costs to the healthcare system including medical management expenses up to $50 billion annually [4], [5]. Among different types of falls, slip-induced falls account for over 30% of outdoor falls and are a major cause of serious injuries among community-living older adults worldwide [6], [7], [8], [9]. Multiple intrinsic factors, such as ineffective stepping, gait disorders, decreased agility, muscle weakness, and cognitive impairment contribute to increased slip-induced fall risk [10], [11]. Various training paradigms targeting key causative fall risk factors have been developed to reduce the incidence of falls and associated injuries, such as task-specific interventions like multi-directional stepping (targetting rapid stepping and foot placement accuracy) [12], [13], agility training (targetting quick coordinated movements) [14], and dance-based exercises (targets foot placement accuracy and cognitive-motor function) [15], [16]. Such training paradigms have mainly shown improvements in volitional balance control (the ability to maintain balance during voluntary tasks) reported as improvements in outcomes of the Berg Balance Scale (BBS) and walking speed via stepping training [12], [13], improvements in performance on the 30-sec Chair stand and test Timed-Up and Go (TUG) test via agility training [14], and improvements in walking speed and performance on the situp-and-go and Romberg test via dance-based exercises [15], [16]. Alternatively, an emerging task-specific intervention is perturbation-based balance training (PBT), which involves repeated exposure to unexpected perturbations to trigger rapid reactions to regain postural stability. PBT has shown improvements in volitional (TUG, BBS, and Mini-BESTest) [17] and reactive balance control (reactive stability, reactive stepping) [18], thereby reducing slip-induced fall risk among older adults [19], [20]. Further, previous studies have shown a > 50% decrease in falls incidence over 1-year follow-up period with slip-PBT [21], [22]. Slip-PBT exposes individuals to repeated forward external base of support (BOS) disturbances that mimic real-life slips in a safe environment. Such training has been implemented using slippery contaminants, or motorized treadmills, or overground movable platforms [23], [24], [25], [26].

Overground-slip training via moveable platforms or slippery contaminants both could replicate real-life slips to facilitate the error-driven learning of necessary motor skills for preventing falls [22], [27]. Although the slippery contaminants-induced slips could highly mimic real-life slip slip scenarios, it is challenging to control the slip characteristics (slip velocity and direction) and maintain consistent slip conditions, which can affect the reproducibility and reliability of the training. Whereas, movable platforms are widely used to investigate the effects of overground-slip training. There is extensive evidence that overground slip training during walking induces significant motor adaptations in post-slip (reactive) COM state (i.e., its position and velocity) stability leading to a rapid reduction in laboratory induced falls (from ~50% to 0%) within a single session of training [22]. Such changes in stability are known to occur from both proactive (pre-slip) and reactive (post-slip) alterations in slipping limb kinematics impacting post-slip braking impulse and slip intensity, including displacement and velocity [28]. Additionally, post-slip changes in the compensatory (recovery) stepping response further contribute to these stability changes [29]. These biomechanical changes are known to be retained for up to a year [22]. Motor adapatations to external perturbation might result from changes in neuromuscular activations induced by neural adaptations within the central nervous system [30], [31], probably resulting from acquistion of new motor strategies [32]. This hypothesis could be substantiated by evidences. First, distinct muscle synergies involving structures or coactivation patterns have shown to be recruited during perturbed versus unperturbed overground gait and between falls and recoveries [33], [34]. Second and more importantly, repeated slip-induced motor adaptation results from the generation of new muscle synergies and discarding redundant synergies [32]. Such neuromuscular adaptive changes were accompanied by changes in joint torques and associated with improved recovery outcomes [32].

Although there is significant mechanistic and behavioral evidence on the efficacy of slip-PBT using overground movable platforms, such paradigms requires a large space and a complex customized setup for an instrumented-walkway, thus limiting applicability to clinical and community settings. Alternatively, devices like motorized treadmills are commercially available, require less space and could be more feasible for clinical and community implementation. Treadmill-slip training can be delivered in stance or walking via a forward displacement of the BOS (treadmill belt) relative to the body’s center of mass. This training has demonstrated efficacy in enhancing an individual’s fall threshold (the perturbation intensity leading to a fall), compensatory stepping response, and reactive stability. Moreover, slip training based on single-belt treadmill has shown to reduce real-life falls, improve clinical balance measures and enhance falls self-efficacy in young [35], older adults [36] and people with stroke [37], [38]. Although many studies have used single-belt treadmills for stance slip-PBT [18], [39], [40], [41], split-belt treadmills are also increasingly being utilized, as it might deliver real-life like perturbations by specifying velocity profiles for each belt. Hence, split-belt slip training can be used to evaluate the effect of PBT on reactive balance control and compensatory responses [42]. Along these lines, previous studies using split-belt treadmill training investigated the joint moments of the compensatory limb during the first stepping response following an unexpected slip perturbation induced by a split-belt treadmill [43] and examined the key factors affecting balance control [44], [45], [46]. Split-belt treadmill PBT also reported successful motor adaptation with improvements in proactive and reactive balance control with repeated perturbations [47], [48], suggesting treadmill-slip training is effective for fall prevention. However, split-belt treadmills are typically more expensive, require more space, are more difficult to design the slip profile, and participants may require additional training to adapt to walking on them. Therefore, single-belt treadmills were used in this study.

To explore the factors contributing to the effectiveness of treadmill PBT, previous studies have examined the biomechanical variables during single-belt treadmill-slip training in stance among healthy young and older adults [18], [39], [40]. One session of treadmill-slip PBT showed reduction in laboratory falls compared to the control group receiving unperturbed treadmill walking in both healthy young (28.6% vs 55.0%) [36] and older adults (9.6% vs. 43.8%) [41]. Such reduction in fall incidence was attributed to improvements reactive COM stability [39], [40] and enhanced compensatory stepping responses [18]. Participants in the training group shifted their COM more anterior relative to the BOS than the control group, therefore, less forward momentum was required to enable the COM to catch the BOS after slip onset, thus reducing the possibility of backward falling. Additionally, the training group more quickly executed the compensatory step, which could help reestablish the BOS and regain stability, further reducing the likelihood of falls [41]. Such adaptational changes observed in biomechanical variables are postulated to occur from CNS-driven changes in neuromuscular units (e.g., muscle activations, latencies, or muscle synergies) [49], [50]. Muscular coordination is achieved via networks of local connections between neurons that reside at a number of sites in the CNS and motor training could influence manner of muscle recruitment during functional tasks [51]. Studies have also shown that the sensorimotor cortex is involved in the modulation of muscle synergies [52], [53]. With repeated perturbations, the CNS is postulated to update motor commands to minimize discrepancies or errors between expected and actual postural states to improvise and subsequently execute optimal neuromuscular responses for balance recovery [32].

Although the kinematic and kinetic factors contributing to motor adaptations during treadmill-slip training have been well examined, the underlying neuromuscular mechanisms are still not understood. Only a handful of studies have examined the muscle activations and onset latencies during treadmill-slip training [54], [55]. Results from these studies have provided valuable information about individual muscle responses. For instance, studies have shown reduced knee coactivations, and higher hamstring activations among healthy adults, which could produce higher force in hamstring muscles, decelerating the forward leg momentum before heel contact and reducing the heel contact velocity pre-to-post slip training, thereby lowering the fall risk [49]. However, it is still unknown how groups of muscles work together to improve balance control during treadmill-slip training. In daily tasks, most movements arise from the coordination of a number of muscles acting together as functional synergists. The movement performance is therefore determined by both intramuscular factors and muscular coordination as they simplify the CNS demands by reducing the number of variables that need to be managed simultaneously [51]. Therefore, muscle synergies represent the most fundamental tier of the motor control hierarchy and may serve as a means for understanding the intricate execution patterns of muscle activity [56]. Muscle synergies can be extracted from recorded electromyography (EMG) signals for a group of muscles. Examining changes in muscle synergies with repeated treadmill-slip could provide an understanding of neural strategies for muscle coordination related to slip adaptation.

To our knowledge, no study has investigated the changes in muscle synergies of lower limbs during treadmill-slip adaptation. It could be hypothesized that repeated treadmill-slip training results in alterations in the structure of muscle synergies via changes in specific muscle(s)’ contribution (or weight) within the recruited synergies (known as reweighting). Previous studies found that stroke survivors could improve motor function by reweighting the activities of specific muscles but not by changing entire synergies [57]. Our previus study also found reweight of muscle syneriges occured duirng overground-slip training for fall prevention [32]. These refined synergies may also play a crucial role in enhancing reactive responses during treadmill-slip training, and the recrutiment of these key muscle synergies might account for the training induced adaptations observed in kinematic variables previously [18], [41]. Examining muscle synergies during treadmill-slip training might have some implications. First, this study could inform the research and clinical community about the previously unexplored neuromuscular changes underlying motor adaptation to treadmill-slip training. Second, identifying key muscle synergies during treadmill-slip training in young adults could assist in future comparison with populations at high fall risk. Further, identifying the pattern of changes in muscle synergies could potentially assist in evaluating the effectiveness of treadmill-slip training protocols. For example, if no specific changes are observed in the muscular synergies with repeated slips, might suggest a need for modification in the intervention protocol components (e.g., intensity and frequency).

The present study aimed to examine changes in neuromuscular control during repeated treadmill-slip training among healthy young adults. Previous studies have shown motor adapation during *stance* treadmill-slip training in different populations including heatlhy young and older adults [18], [58]. Therefore, as a first step to investigate changes in neuromuscular control during motor adaptation, we analyzed muscle synergies during a block of repeated stance-slips among healthy young adults. Based on previous evidence indicating that motor adaptation to slips might be related to generation or elimination of muscle synergies [32], we hypothesized that there would also be changes in the composition of muscle synergies from the early-training stage (the first few slips) to the late-training stage (the last few slips) during treadmill-slip training.

## II. METHODS

### A. Subjects

Thirty healthy young adults were recruited in this study, they were screened via a customized telephone screening questionnaire before the experiment to exclude individuals with any neurological, musculoskeletal, cardiopulmonary, or other systemic disorders and recent surgeries or hospitalizations. Three were excluded for the data collection due to recent fracture and larger body weight exceeding the load capacity of the full-body safety harness (> 250 lbs.), and one were excluded for the data analysis due to persistence of artifacts in collected data. The exclusion criteria were set in place for participant’s safety and to ensure a homogenous sample. Twenty-six were included in this study, including 11 males (age: 28.7±5.2 years; height: 173.8.9±8.4 cm; weight: 80.7±17.4 kg) and 15 females (age: 27.4±3.3 years; height: 161.8±5.8 cm; weight: 64±13.5 kg). The study was carried out in accordance with the Declaration of Helsinki of 1975, and all participants provided written informed consent before the slip experiment. The experimental protocol and informed consent were approved by the Institutional Review Board of the University of Illinois at Chicago (#2017–1069, date of approval: 9 February 2018). This study is registered at ClinicalTrials.gov (“Reactive Balance Training for Fall Prevention”, NCT04205279).

### B. Experimental Protocol

**Fig. 1.**
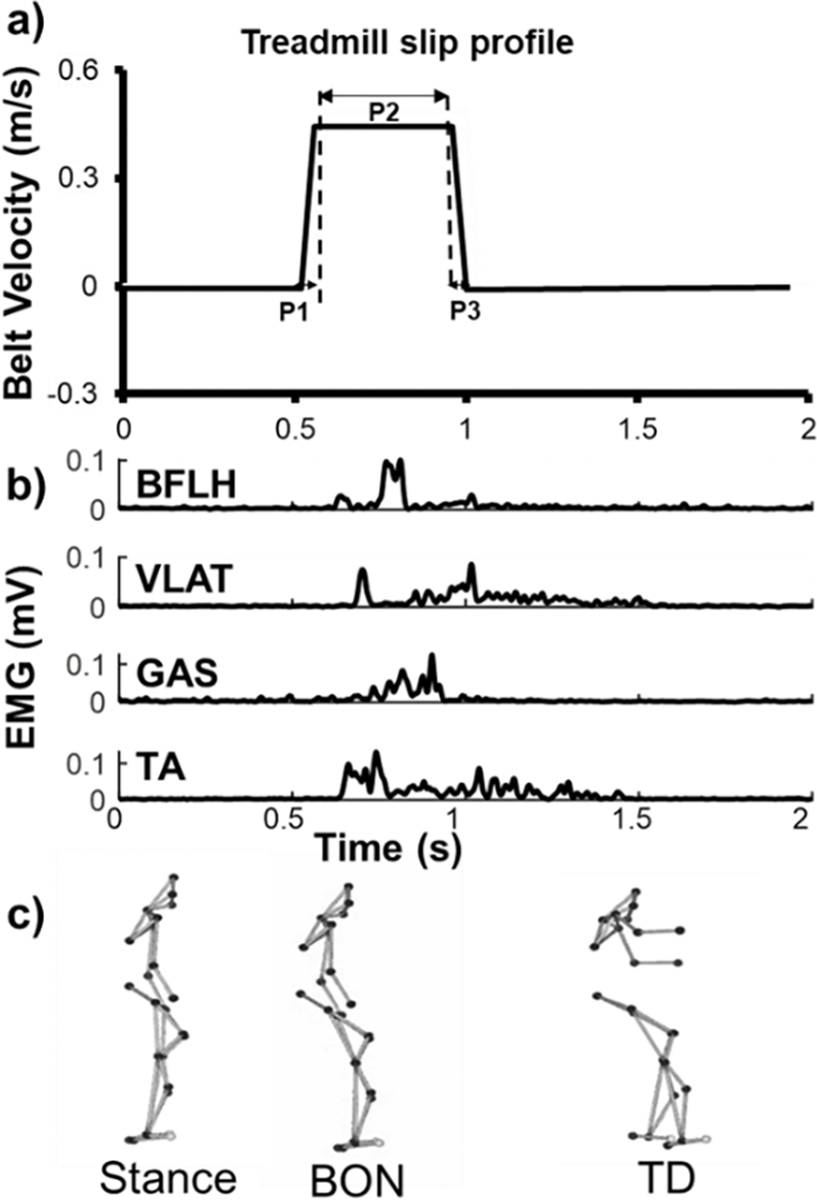
a) The treadmill profile for the stance treadmill-slip training. The profile contains three phases: P1: acceleration phase, P2: constant speed phase and P3: deceleration phase. b) the representative rectified EMG signals for the recovery limb muscles following slip perturbation, including biceps femoris long head (BFLH), vastus lateralis (VLAT), medial gastrocnemius (GAS), and tibialis anterior (TA). c) the schematic representation of a reactive stepping in this study at the time events of stance still, belt onset (BON), and recovery touchdown (TD).

All participants received 10 repeated slip-like perturbations during stance triggered by ACTIVESTEP motorized treadmill (SIMBEX, Lebanon, New Hampshire). We selected 10 trials based on a previous study showing adaptive changes in reactive performance in the first 10 treadmill-slip trials and the following trials resulted in limited improvements, which could be considered as a plateau phase [18]. In this study, participants were exposed to sudden, slip-like treadmill perturbations without prior knowledge on the exact onset time of perturbation. Participants were told that a slip-like disturbance could occur at any given time, and that upon experiencing the slip they had to try their best to recover their balance without falling. Perturbations were triggered at a random instant within 2–4 secs after the start of each trial (indicated by a “START” sound). A familiarization trial (Belt displacement: 0.14 m, velocity: 0.36 m/s, acceleration: 9 m/s^2^) was first given at a lower intensity to minimize the effect of the startle response. The treadmill-slip training trials were subsequently given at a higher intensity with belt displacement of 0.18 m, velocity of 0.45 m/s, and acceleration of 11.35 m/s^2^ and trial duration of 8 seconds (Fig. 1a). The intensity for both the familiarization and slip training trials were chosen based on the protocol from previous stance perturbation studies showing that perturbation intensity was strong enough to induce motor adaptation [18], [59]. The slip perturbation occurred when the treadmill belt translated forward resulting in a backward loss of balance, and all participants took at least one recovery step to prevent a fall. As previous study reported that fall prevention mainly rely on the reactive responses before and at recovery touchdown (~400 milliseconds - ms after belt onset) [60], therefore, a slightly longer duration (480 ms) was chosen to ensure adequate coverage to improve the reactive response using slip training considering possibility of individual variability. There was a rest interval of 45–60 seconds (s) between the consecutive slip trials, allowing participants to relax and reset their body during this rest period. All participants were informed about the study procedures and experimental risks during the informed consent process.

### C. Data Collection and Processing

Participants were equipped with a full-body safety harness which was connected by shock-absorbing ropes to a load cell (Transcell Technology, Inc., Buffalo Grove, IL). The ropes were adjusted for each subject so that should they fall and suspend from the track after slip occurrence, their palms, knees, and buttocks would not contact the walking surface. After adjustments of the rope lengths, every subject was asked to perform a standard sitting-in-harness trial for 8 s to ensure their lowest hip height is around 35% of body height in this trial [61]. A fall was identified if the peak load cell force measured during a slip exceeded 30% of the participant’s body weight. This threshold was established and verified by a previous study for both young and older adults, with an accuracy of 100% [61]. Kinematic data was collected using an eight-camera motion capture system (Qualisys AB, Gothenburg, Sweden). Twenty-six markers from the Helen Hayes marker set were placed on bilateral bony landmarks, the head, and the trunk to compute the joint centers [62], three markers were placed on the ground and one on treadmill belt. The raw marker data was low-pass filtered using a fourth order Butterworth filter with a cutoff frequency of 6 Hz [63], and the kinematic variables were computed using customized MATLAB routines (MATHWORKS, Natick, MA). The kinematic data was collected at 120 Hz and synchronized with loadcell and electromyography (EMG) data at 600 Hz.

DELSYS Trigno surface EMG sensors were used to record EMG activity with signals sampled at 600 Hz. EMG activity was recorded from four muscles including the tibialis anterior (TA), medial gastrocnemius (GAS), vastus lateralis (VLAT), and biceps femoris long head (BFLH). Raw, unrectified EMG signals were first hardware band-pass filtered with a bandwidth of 20–450 Hz, and a standard mode rejection ratio of > 80 decibels applied. After data collection, the EMG signals were digitally high-pass filtered over 35 Hz and then full-wave rectified (Fig. 1b). The rectified signals were then smoothed via a second-order, dual-pass Butterworth low-pass filter with a 40 Hz cutoff frequency [64].

### D. Outcome Variables

To investigate the motor adaptation during treadmill-slip trials, both kinematic variables and muscle synergies were compared between the early-training stage and the late-training stage. The first two slip trials (S1-S2) and last two trials (S9-S10) during repeated slip training were selected to represent the early and late-training stages, respectively.

Kinematic variables: We assessed variables including reactive recovery stepping responses (step initiation and execution times, and step length), and COM position and velocity, and trunk angle. All these kinematic variables were calculated in the sagittal plane and have been previously used to measure motor adaptation in balance recovery responses [28], [65]. The crucial time events chosen were belt onset (BON), recovery foot liftoff and its touchdown (TD) during stance treadmill-slip trials (Fig. 1c). The events of liftoff and TD were determined from heel marker coordinates in the anteroposterior direction [28]. BON was defined as the time when the velocity of the belt marker exceeded 0.05m/s in anteroposterior (AP) direction [66]. Step initiation time was the duration from BON to recovery (stepping) foot liftoff, and step execution time was the duration from recovery foot liftoff to TD. The total duration analyzed in this study was between BON-100ms and TD+100ms, which contains both the proactive phase (i.e., prior to BON) and reactive phase (i.e., after BON). Step length was calculated as the distance between the slipping heel and recovery heel at TD in AP direction. Step width was calculated as the distance between these heels at TD in mediolateral (ML) direction. AP COM position was calculated as the distance between the projected COM location (estimated using a 13-segment model) [67] and recovery heel at TD in AP direction, and then normalized by length of BOS (distance from trailing heel to leading toe in AP direction). AP COM velocity was calculated by subtracting the velocity of recovery foot (heel) from the velocity of COM at TD in AP direction. ML COM position was calculated as the smaller distance from the projected COM location at TD to both left edge and right edge of the BOS [68], and then normalized by the width of BOS [distance between the two toes (fifth metatarsal) in ML direction]. ML COM velocity was calculated as the absolute velocity in ML direction as the BOS velocity in ML direction is 0 m/s. Trunk angle relative to transverse plane was calculated using the midpoint of shoulder markers and midpoint of hip markers. A value < 90 degree indicates trunk flexion and a value > 90 indicates trunk extension. Arm angle relative to vertical line was calculated using shoulder marker and elbow marker for both arms, and the one with larger flexion angle at TD was reported.

Muscle synergy extraction: EMG data for 8 muscles from pre-BON (BON – 100ms) to post-TD (TD + 100ms) was down-sampled by averaging the data in 10 ms bins, and then EMG data from consecutive trials (S1-S2, or S9-S10) were concatenated end-to-end to create matrices that were 8 (number of muscles) × n (number of time bins) in size [34]. S1-S2 were used for muscle synergy extraction for early-training stage, and S9-S10 were used for late-training stage. The concatenated trials were used to enhance precision and reliability in the extracted muscle synergies [69]. The 10 ms time bins were selected to have sufficient time resolution and to avoid dealing with point-to-point changes in the highly variable EMG signals [70]. The concatenated EMG matrices were then normalized to the maximum activation in all the trials [71], and each row (muscle vector) was scaled to have unit variance to ensure that each muscle was equally weighted in the extraction. This unit variance was removed after muscle synergy extraction, changing the muscle synergies back to their original scaling.

Muscle synergies were extracted individually for each participant from EMG matrices with unit variance for early-training and late-training stages using our customized MATLAB routines [72]. We employed non-negative matrix factorization in MATLAB (nnmf.m), which is a decomposition algorithm used extensively in muscle synergy analysis [34], [71], [73]. This algorithm assumes that a muscle activation pattern: M evoked by a perturbation in each period comprises of a linear combination of a few muscle synergies: $w_{i}$, which are each recruited by a synergy recruitment coefficient: $c_{i}$. Therefore, a particular muscle activation pattern would be characterized by the following equation: $M=c_{1}w_{1}+c_{2}w_{2}+\ldots +c_{n}w_{n}+\varepsilon$.

In this equation, *ε* is a scalar of EMG noise, $w_{i}$ is a vector corresponding to the ith muscle synergy, and each $w_{i}$ is multiplied by a scalar recruitment coefficient, $c_{i}$. The spatial components were considered as a fixed time-invariant pattern, while the temporal activation coefficients varied across time [56]. Therefore, activation represents how the group of muscles in $w_{i}$ is activated over time. To compare the temporal activation coefficients among different stages, these coefficients in the whole duration (pre-BON to post-TD) were scaled to same length for each subject with 100 points.

The number of muscle synergies required to explain any of these datasets was determined by selecting the smallest number of synergies that could adequately reconstruct the muscle responses, which was quantified by the variance accounted for (VAF). To ensure consistency within each condition, the number of muscle synergies selected was the minimum number at which the muscle synergies that accounted for greater 90% of the overall VAF [64]. A local criterion that the individual VAF for each muscle was above 75% [64] and subsequent increase of the number of synergies did not give more than 5% increase in the mean value of these individual VAFs was imposed [74].

Due to the individual variability in neuromuscular control, there might be a broad range of data across the individual synergies, to facilitate comparison of muscle coordination patterns between stages, muscle synergies extracted from each condition were first pooled across participants and grouped with a k-means cluster analysis to minimize the sum of squared Euclidean distances of each synergy with respect to the centroids [34]. Such a method could identify synergistic patterns characterizing all participants and reduce the effect of the innate variability of the muscle activity on the extracted motor patterns. The number of clusters for each condition was determined as the smallest number that yielded a local maximum of average silhouette value (across 1 to 8 clusters). Each run of the k-means algorithm was repeated 100 times to ensure robustness of the cluster number.

### E. Statistical Analysis

To investigate the kinematic differences between the early- and late-training stages, we first used the Kolmogorov–Smirnov test to check for normality of data. This was followed by a repeated measures ANOVA to examine the training effect on COM position, COM velocity, step length, step width, initiation time, execution time, trunk angle and arm flexion across S1, S2, S9 and S10. A Paired-samples t-test was also used to compare the average values for these variables between the two stages, the S1-S2 were averaged for early-training stage analysis and the S9-S10 were averaged for late-training stage analysis.

To test the differences in muscle synergies between the early- and late-training stages, the dimensionality (number) of muscle synergies was first compared using a paired Wilcoxon signed rank test. Next, the correlation coefficient (r) was calculated between the average muscle synergy vectors (in each cluster) between different stages. A pair of muscle synergy vectors (degree of freedom is 6) were considered to be high similarity if they had r > 0.83, which represents statistically significant similarity (p < 0.01) [75]. The muscle synergies from different stages with significant similarity were classified into the same mode. Further, the weight of each muscle in the synergy (muscle components in vector $w_{i}$) was compared between early- and late-training stages within each mode using dependent t-test to check the specific changes in the structure of muscle synergies. For the temporal activation $c_{i}$, the peak value of activation amplitude, the area under the activation curve, the time of peak activation, and time of activation onset (instant when activation > 25% peak activation [76]) were also compared between stages for each mode using dependent samples t-test. Statistical significance was assumed at p < 0.05.

### F. Sample Size Justification

Sample size was estimated using G* power software (G* Power 3.1) from preliminary data (n = 10), which yielded medium to larger effect sizes [Cohen’s d (*δ*): 0.55–1.17] for primary outcomes. Preliminary data used for sample size estimation was also included in the data analysis. With expected effect sizes, an estimated sample of 20 participants were obtained to achieve > 80% power. Previous muscle synergy studies have also demonstrated that a sample of 20 participants are appropriate to obtain significant results [77], [78], hence we enrolled 26 participants in this study. To quantify the strength of the difference in the key outcomes between early- and late-training stages, the effect size (*δ*) of our results based on 26 participants was also calculated.

## III. RESULTS

**Fig. 2.**
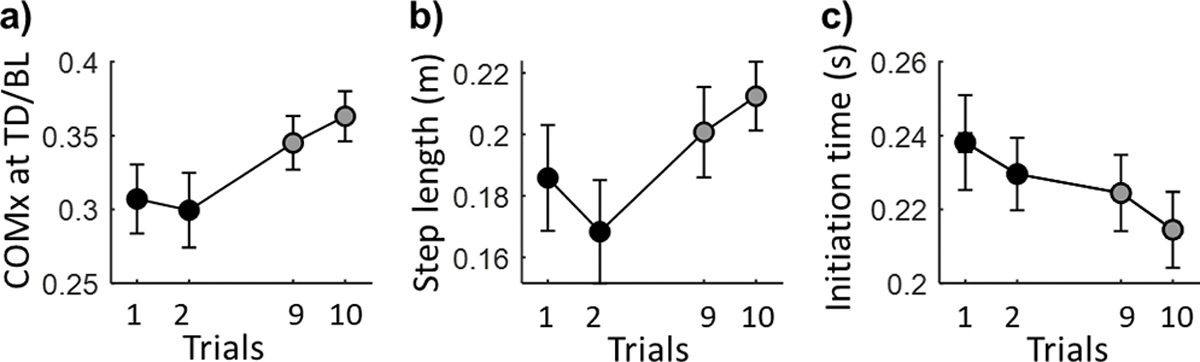
The mean and standard error of kinematic variables with significant training effects for early-training stage (S1-S2 marked as black circle) to late-training stage (S9-S10 marked as gray circle). COMx denotes COM position, BL denotes BOS length, TD denotes the recovery foot touchdown.

All 26 participants took a backward step in both early-training stage (S1-S2) and late-training stage (S9-S10), but none of them fell in these slip trials. Among all the kinematic variables, training effects were detected in COM position [F(3, 75)= 2.77, p < 0.05, Table I], step length [F(3, 75)= 2.73, p < 0.05] and initiation time [F(3, 75)= 4.04, p < 0.05] using repeated measures ANOVA. Specifically, the average initiation time became 0.03s shorter (0.25 vs. 0.22s, p < 0.05), the step length became 0.03 longer (0.17 vs. 0.2 m, p < 0.05) and the COM position at TD was more anterior (0.31 vs. 0.35, p < 0.05) after receiving the treadmill-slip training. The trial-to-trial changes in these kinematic variables with signiciant training effects were showed in Fig. 2.

**Fig. 3.**
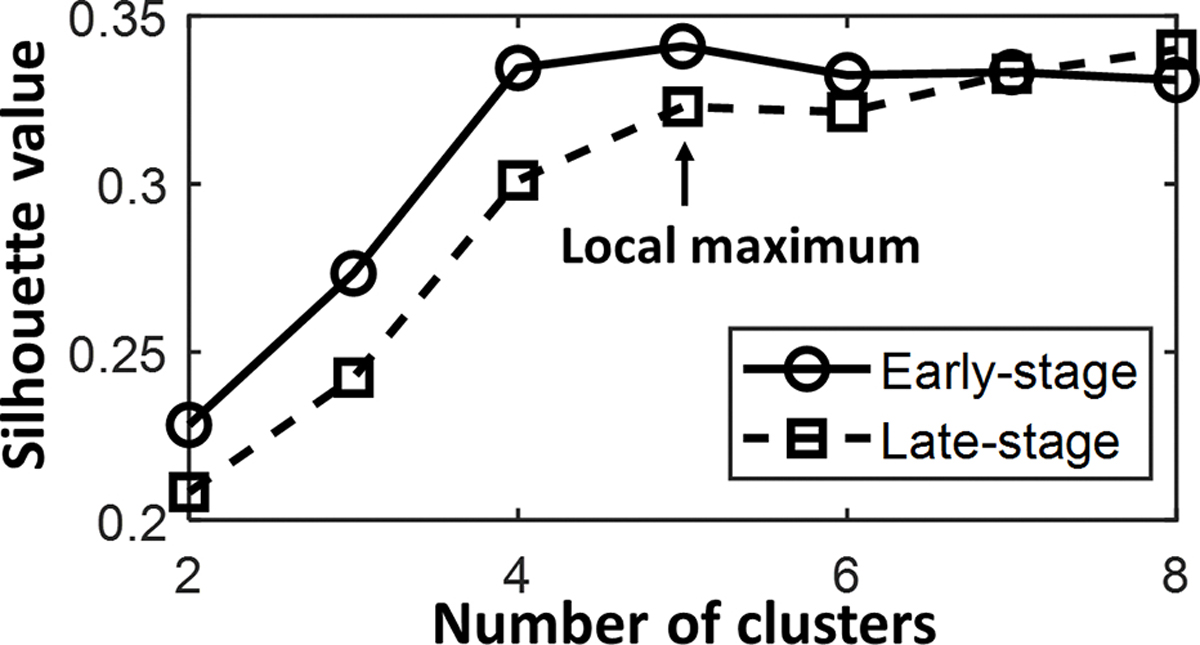
The average silhouette value for different number of clusters in early-training stage and later-training stage, the local maximum appeared when number of clusters was 5 for both stages.

Consistent to kinematic variables, the dimensionality of extracted muscle synergies was similar between the early- and late-training stage (p > 0.05, Table II). The dimensionality ranged from 3 to 5 in early-training stage, and ranged from 3 to 6 in late-training stage, while only 2 out of 26 participants recruited the 6 synergies (dimensionality = 6). Clustering results showed that 5 different modes of synergies were recruited in both the early- and late-training stages (Fig. 3).

**Fig. 4.**
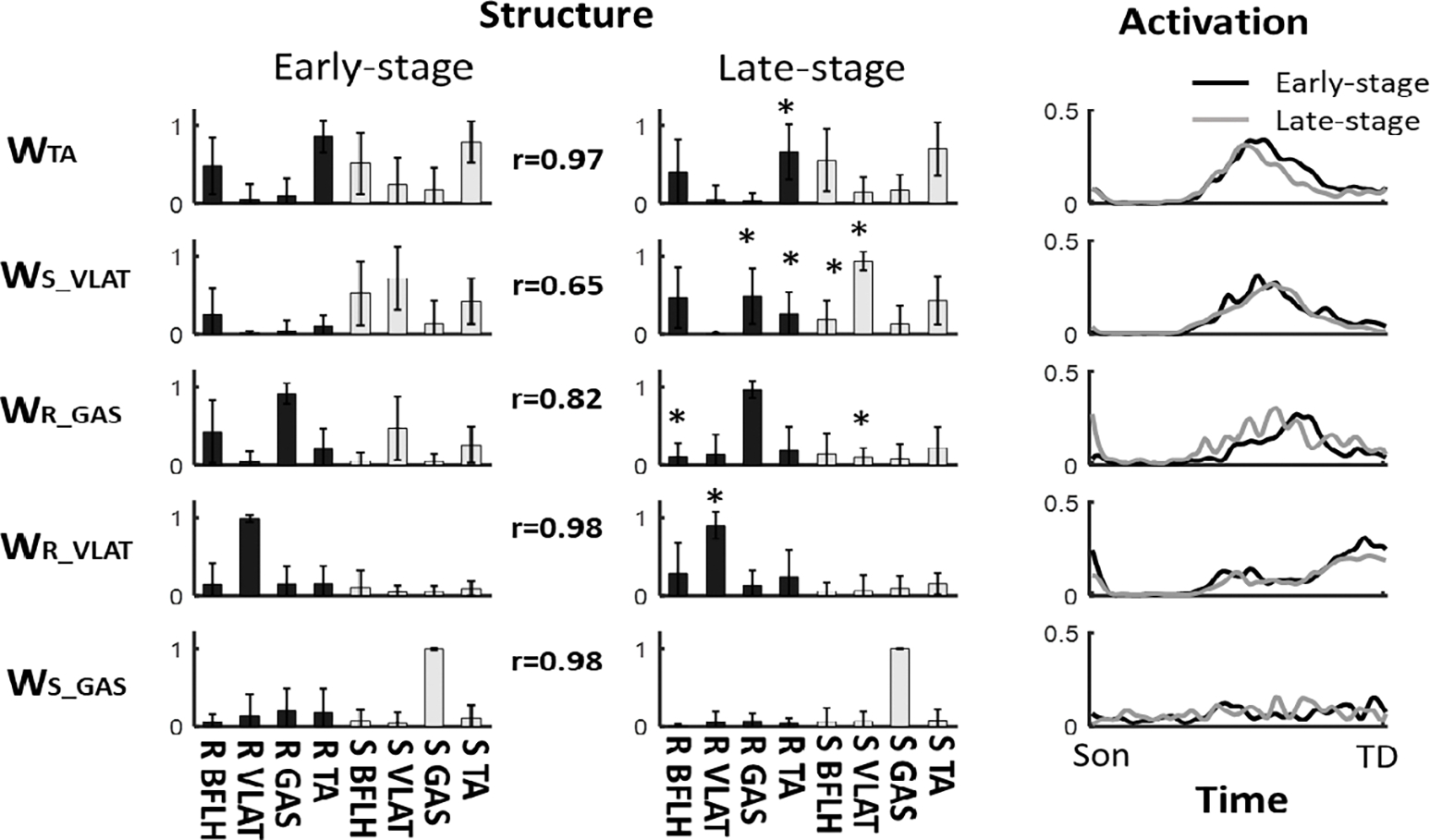
Comparison of structure and activation for clustered synergies between early- and late-training stages. The clustered synergies were ordered by the time of peak in the activation curve from slip onset (SON) to recovery touchdown (TD) and were named by the dominant muscle(s). Here, R denotes recovery side, S denotes slipping side, TA denotes tibialis anterior, VLAT denotes vastus lateralis, GAS denotes medial gastrocnemius, and * denotes p < 0.05.

We named the modes by the dominant muscle(s) and ordered by the peak time of activation curve **(W**_**TA**_**, W**_**S_VLAT**_**, W_R_GAS_, W**_**R_VLAT**_, and **W_S_GAS_).** Among these different synergy modes, **W**_**TA**_**, W**_**S_GAS**_, and **W**_**R_VLAT**_ showed a high similarity between early- and late-training stages (r > 0.97 for all, Fig. 4), **W_R_GAS_** showed a medium similarity (r = 0.82), and W_S_VLAT_ showed a low simialrity (r = 0.65) between the two stages.

**Fig. 5.**
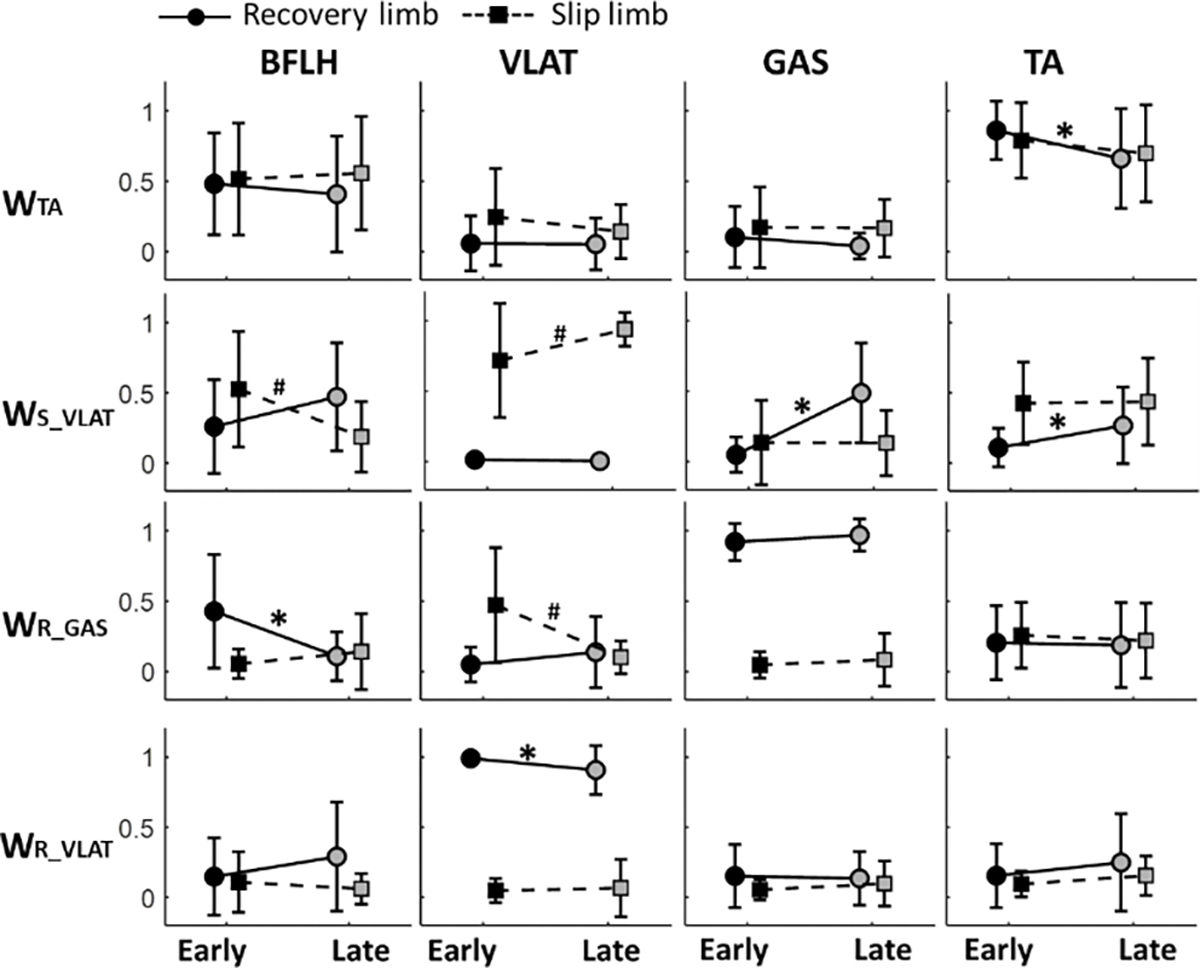
The muscle weight comparison between early-training and late-training stages for synergy modes **W**_TA_(Tibialis anterior dominant), **W**_S_VLAT_ (Slipping side Vastus Lateralis dominant), **W**_R_GAS_ (Recovery side Gastrocnemius dominant), and **W**_R_VLAT_ (Recovery side Vastus Lateralis dominant). Here, **W**_S_GAS_(Slipping side Gastrocnemius dominant) was not included as no significant difference in muscle weight were found between stages. * denotes p < 0.05 for recovery limb, and ^#^ denotes p < 0.05 for slipping limb.

In synergy mode **W**_**S_GAS**_, no difference was found in the weight of the muscles, the recovery VLAT (*δ* = 0.55) in **W**_**R_VLAT**_ and the recovery TA (*δ* = 0.71) in **W**_**TA**_ was lower in the late-training stage than the early-training stage (p < 0.05 for both in Fig. 5). In **W**_**R_GAS**_, both the recovery BFLH (*δ* = 0.97) and slipping VLAT (*δ* = 1.06) reduced after receiving the slip training (p < 0.01 for both). Whereas, 4 out of 8 muscles in W_S_VLAT_ showed singificant changes from early- to late-training stage (0.64≤ *δ* ≤1.47, p < 0.05 for all). Regarding the temporal activation for these synergy modes, the area of activation in **W**_**TA**_(*δ* = 0.6), the peak value of activation in **W**_**R_VLAT**_ (*δ* = 0.89) and the activation onset in **W**_**R_GAS**_ (*δ* = 1.01) showed a reduction from early- to late-training stage (p < 0.05, Table III).

**Fig. 6.**
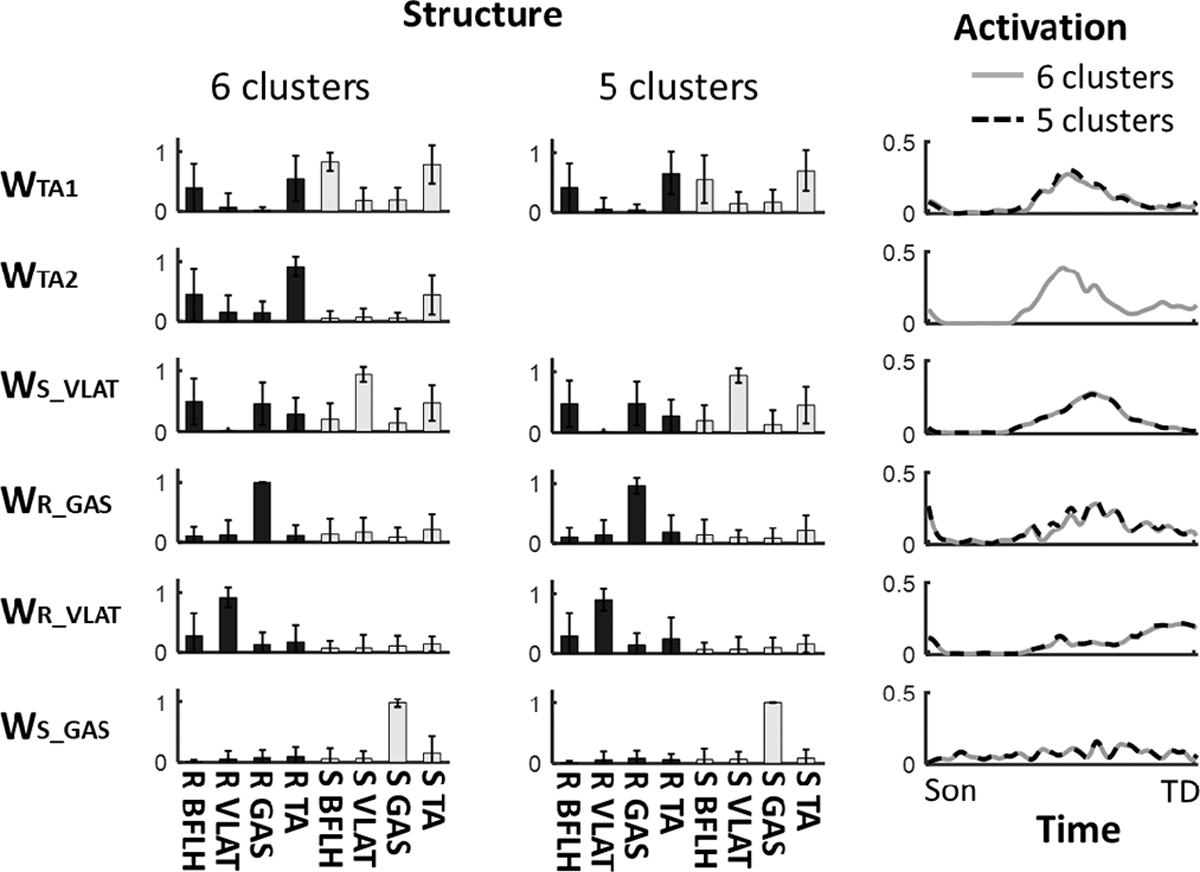
Comparison of structure and activation for clustered synergies in late-training stage between different clusters (5 vs. 6) using k-means cluster analysis. All clustered synergies showed a high similarity (r > 0.99) in structure and activation curve between 5-cluster and 6-cluster methods except W_TA_ (W_TA1_: r = 0.94, W_TA2_: r = 0.66). Here, R denotes recovery side, S denotes slipping side, TA denotes tibialis anterior, VLAT denotes vastus lateralis, GAS denotes medial gastrocnemius.

For the synergies in the late-training stage, the silhouette value is similar between 5-cluster and 6-cluster, and the maximum synergy dimensionality in this stage is 6, hence, the synergies in this stage were also clustered as 6 modes and compared with the 5-cluster results. The comparsion showed that the structure and activation cruve was highly similar between these two clustering methods (r > 0.99 for **W**_**S_VLAT**_, **W**_**R_GAS**_, **W**_**R_VLAT**_, **W**_**S_GAS**_ in Fig. 6). The 6-cluster method resulted in another two synergy modes, which recruited both TA and showed similar activation curve, hence, they were named as **W**_**TA1**_ and **W**_**TA2**_. **W**_**TA1**_ is similar to **W**_**TA**_ in Figure 3 (r = 0.94), while **W**_**TA2**_ showed a low similarity of 0.66.

## IV. DISCUSSION

This study examined the neuromuscular changes during repeated exposure to a block of treadmill-slips during stance among healthy young adults. The findings showed young participants demonstrated training-induced adaptations in neuromuscular control (changes in structure and activation of the muscle synergies) reflected by kinematic changes in variables related to balance recovery. These results partially supported our hypothesis that treadmill-slip training could induce alterations in the composition of muscle synergies.

Stance treadmill-slip training induced adjustments in the structure of muscle synergies mainly on the *recovery (stepping) side* in this study. These changes in muscle synergies could explain the improvements observed in recovery stepping response (e.g., longer step length). Such changes in recovery stepping responses have shown to induce successful motor adaptation to treadmill-slips among young adults [18]. In this study, four (**W_TA_, W_S_VLAT_, W_R_GAS_, W_R_VLAT_)** out of five synergies showed significant changes in the weight of muscles on the recovery side (Fig. 4). Specifically, we observed a reduction of the muscle weight in **W**_**TA**_**, W**_**R_GAS**_, and **W**_**R_VLAT**_ and an increment in the ankle muscle weight (TA and GAS) of the recovery side in **W**_**S_VLAT**_ from early- to late-training stage. This increment in the weight of ankle muscles (especially in GAS) might have helped in shortening recovery step initiation time, as GAS plays a crucial role in generating propulsive impulse (force-time integral of the anterior ground reaction force), which is essential for pushing off the ground during step initiation [79]. Although GAS of the recovery side in **W**_**R_GAS**_ was also highly activated (weight close to 1) in both stages, the timing of peak activation in **W**_**R_GAS**_ was later than W_S_VLAT_ in the early-training stage (Fig. 4). This indicates that **W**_**R_GAS**_ might be recruited for adequate foot landing during the late-swing phase in the early-training stage, but not for step initiation. Along with the reduction in the weight of BFLH on the recovery side, W_R_GAS_ might have assisted in quick landing with larger knee extensor torque in the late-training stage, as time of rapid stepping was previously shown to be strongly correlated with peak knee extensor torque [80]. **W**_**R_VLAT**_, which peaked at the end of the activation curve (close to TD), might have provided the propulsive impulse after recovery TD. The larger weight in this synergy structure and higher peak activation in the early-training stage could have led to a larger propulsive impulse applied on the COM to avoid balance loss [28]. On the other hand, a larger propulsive impulse might also reflect an overcompensation to the slip perturbation, indicating that an excessive but not optimal impulse was applied for balance control in the early-training stage. The weight in **W**_**R_VLAT**_ reduced treadmill-slip training possibly to avoid an overcompensation. Previous study also found that this overcompensation reflected by early proximal muscle activation during perturbed walking and these muscle activation in the initial slip trials tends to fade away in following slip trials [81]. Similar changes were found in the activation of **W**_**TA**_ from early- to late-training stage (muscle weight in Fig. 5 and area in Table III). These reductions in muscle activation could probably minimize the muscle effort and metabolic energy expenditure without affecting the reactive performance [82]. Thus, it seems that CNS optimizes muscle synergies by modifying the weight of specific muscle(s) during treadmill-slip training rather than generating new synergies (represented by low similarities between pre- and post-training).

On the *slipping side*, only **W**_**R**_**_**_**GAS**_ and **W**_**S**_**_**_**VLAT**_ showed changes in the weight of muscles. The lesser changes seen on the slipping side might be influenced by the nature of the slips triggered by motorized treadmills. Motorized treadmills allow customization of perturbation parameters (displacement, velocity, and acceleration), but these preset parameters cannot be modulated by the slipping limb. Therefore, the adjustments of the muscle synergies on the slipping limb might have no effect on the slip intensity, resulting in modest improvement in COM stability. It is known that a slip perturbation induces a motor error, which is the discrepancy between predicted performance and actual performance. According to the principles of motor learning (i.e., feedback-error-learning) [83], [84], [85], [86], [87], this motor error prompts adjustments to locomotor commands to counteract the slip perturbation [85], [86], [87], which could result in modifications in muscle synergies on both limbs following exposure to the novel slip. However, if the adjustments in motor commands to regulate slip intensity prove ineffective in influencing slip performance in the subsequent slips, those motor commands along with resulting muscle synergies may be disregarded by CNS. Consequently, fewer changes in the weight of slipping limb muscles occurs during the late-training stage. Significant changes in the muscle weights of slipping limb were observed in **W**_**S_VLAT**_, where the weight of VLAT increased and the weight of BFLH decreased in the late-training stage compared to the early-training stage. Although such changes do not affect the slipping intensity, they could enhance the vertical support force by providing larger knee extensor moments. Larger knee extensor moments during a slip perturbation could enhance the limb support, which is established to be the key factor for lowering the risk of balance loss or fall [60].

The muscle synergy and kinematic adjustments suggest that 10 repeated stance treadmill-slips resulted in enhanced reactive balance performance. These findings align with a previous study reported significant improvements in post-slip COM stability in young adults who received a block of stance treadmill-slip training [18]. Unlike overground-slip training, participants receiving treadmill-slip training exhibited lesser improvements in COM stability [28], [88]. Similarly, only modest changes in the structure and activation of muscle synergies were found in this study, with 1 out of 5 synergies showing low similarities (*r* = *0.65*) between stages. In contrast, our previous study on overground-slip training found that over 40% (3 out of 7) of the synergies showed low similarity in the late-training stage compared to the early-training stage. Together, these findings suggest that more significant neuromuscular changes might lead to more substantial kinematic changes during training [32]. As aforementioned, one possible reason for these differences between treadmill-slip and overground-slip training could be the lack of effective slip intensity control strategies in the treadmill-slip training, the lack of slip control also primarily influenced muscle synergy structure on the recovery side. Alternatively, for overground-slip training, our previous study revealed substantial adjustments in synergy structure on the slipping side [32], which could effectively reduce the slip intensity and improving the COM stability. Another reason for this discrepancy could be the difference in the distance between COM and the trailing foot at slip onset. In overground slips, perturbations occur at touchdown of the leading foot, allowing the trailing limb to generate a horizontal (propulsive) force to prevent the COM from moving backward, and the force is positively correlated to the distance between the COM and the trailing foot according to the bipedal inverted pendulum model [89]. However, this distance is much shorter in stance slip, potentially limiting participants’ ability to generate sufficient force for COM stability, leading to smaller improvement in COM stability. Consequently, participants may rely on taking a larger backward step to enlarge BOS, thus enhancing COM stability. Such discrepancy in control strategies might restrict the generalization of training effects from stance treadmill-slip training to overground gait slip.

We also investigated the impact of cluster numbers on clustered synergies for the late-training stage, where the Silhouette value closely matched between 5 and 6 clusters. Clustering individual synergies might enable the identification of common synergy patterns across participants, while the selection of cluster numbers could influence the structure and temporal activation of clustered synergies. Our results suggested that the selection of 6 clusters minimally affected our findings, as all the clustered synergies (or modes) still showed similar changes from early- to late-training stage compared to the 5-cluster method. While, an extra mode (**W**_**TA2**_) emerged due to the increment of cluster number, such mode might be generated from **W**_**TA**_ by reducing the weight of slipping BFLH through treadmill-slip training. However, such alterations in clustered synergies do not influence our study conclusion that treadmill-slip training can induce changes in synergy structure.

The adaptation effects seen in neuromuscular control during treadmill-slip training suggest the CNS’s potential role in modifying existing muscle synergies to undergo the process of reweighting (i.e., changes in muscle coordination patterns) rather than generating new ones through merging or fractionation. Although merging of synergies could reduce task-relevant motor complexity and restrict motor variability following repeated perturbations [90], [91], it could also lead to a loss of fine motor control and precision [71], [92]. Alternatively, reweighting of existing synergies seen in this study might have assisted in achieving optimal control of COM stability and recovery landing following treadmill-slip perturbation. This process suggests the CNS’s ability to adapt and refine motor control strategies by selectively enhancing or diminishing the activation of specific muscles within a synergy. For example, our findings of specific changes in muscle synergies, particularly the reweighting of the GAS and VLAT muscles (i.e., an increase in weight of right GAS in **W**_**S_VLAT**_ and an decrease in weight of right VLAT in **W**_**R_VLAT**_ from early- to late-training stage in Fig. 4), suggest that reweighting of the synergy could enhance reactive responses and possibly reduce energy expenditure [93].

Our study results have some potential implications for research and clinical community. This study suggests the importance of examining neuromuscular control along with biomechanical variables when evaluating and monitoring fall prevention training. Currently, the evaluation and progress monitoring of training protocols is primarily based on behavioral and kinematic motor outputs. However, altered behavioral or kinematic outcomes could be related to underlying differences in muscle coordination patterns. Therefore, assessing kinematic outcome alone might not provide adequate information about the variability in adaptation strategies, which are likely to be mediated by different muscle activation patterns. Muscle synergy analysis might provide additional information about the effectiveness of a training protocol, the progression acquired during training and provide precise assessment approaches. Our results further inform the previously postulated importance of stepping response for balance recovery from environmental perturbations such as slips. Our findings indicated that a single session of treadmill-slip training in young adults mainly induced changes in muscle synergies that modulate kinematic adaptation related to the reactive stepping (recovery side) response. Hence, protocols targeting reactive step training could increase resistance to balance loss and falls from environmental perturbations [94], [95]. It must be noted these findings in young adults might not directly translate to other populations like older adults or people post-stroke, they can serve as a basis of comparison for examining age or pathology-induced alterations in neuromuscular mechanisms following session of treadmill-slip training.

This study has some limitations that must be considered when interpreting its findings. First, we only analyzed young adults in this study. While it is known that locomotor adaptability is not significantly affected by age [85], [96], [97], it is possible that older adults use different neuromuscular strategies for locomotor adaptation than young adults. For instance, the reweighting process in muscle synergies may differ in older adults or individuals with balance impairments due to reduced neuromuscular flexibility. These individuals are likely to exhibit slower motor learning and reduced adaptability compared to healthy young adults, resulting in smaller adjustments in muscle weights. Additionally, they may reweight different muscles, for example, increasing the co-contraction of agonist and antagonist muscles as a compensatory mechanism to enhance stability. Hence, the findings from healthy young adults might not directly translate to other populations. However, previous studies have shown similarities in kinematic mechanisms of adaptation between young, older and neurological populations [96], [97]. Hence, it is possible that other populations might exhibit similar patterns of neuromuscular changes following treadmill-slip training. Future studies need to validate the applicability and generalizability of our findings to other populations. Next, the slips were delivered in stance but not in gait. Hence, the findings of this study may not be applicable to slip perturbations during walking or in real-life. However, regardless of whether a slip occurs during stance or in gait, maintaining COM stability and limb support is crucial to avoid a fall. Once a slip initiates backward balance loss, a compensatory step is required to recover stability and enhance limb support. Therefore, the muscle synergy adjustments related to quick landing and limb support enhancement might also be beneficial for fall prevention in different slip conditions. Future studies could use split-belt treadmill systems to examine neuromuscular adaptations resulting from gait-slip training. Then, we estimated the required sample size based on preliminary data from 10 participants. However, with such a small sample, the effect size estimate may be less precise with greater variability. Despite this limitation, results from a larger sample (n = 26) demonstrated a moderate to high effect size (*δ*: 0.41 to 1.47), suggesting that our chosen sample size is robust to capture meaningful effects. Further, we used moderate intensity of stance-slips (slip distance: 0.18m, acceleration: 11.35m/s^2^), which might not have been sufficient to trigger a fall among young adults (fall rate = 0%). Similar to our findings, previously, it has been shown that young adults could resist an unexpected perturbation even with significantly higher intensity in both forward and backward directions, resulting in a lower fall rate [98]. However, with the lower intensity all young adults still demonstrated a recovery stepping response on the first slip and were able to significantly improve their reactive response and control of their COM post training. Our findings are consistent with findings from a previous study using low intensity treadmill-slip training in young adults [41], [99]. Lastly, we employed a single session training design, however, higher frequency (no. of sessions) of gait-slip training in young adults has shown to induce better adaptation in recovery stepping responses [100]. Therefore, it is possible that higher frequency (multisession) stance treadmill-slip training might result in better training effect. However, such postulation needs to be validated with future studies.

## V. CONCLUSION

A single session of stance treadmill-slip training resulted in neuromuscular changes, as evidenced by the reweighting of the muscle synergies and corresponding kinematic improvements in variables related to balance recovery in young adults. The modification of specific muscle synergies from pre- to post-training seen mainly on the recovery side, mechanistically demonstrates the importance of the reactive stepping response for balance recovery from slip-like perturbations. Further research is needed to compare the neuromuscular changes during treadmill-slip training in populations with high fall-risk to understand age or pathology-related neuromuscular alterations in motor adaptation to reactive balance responses.

## Figures and Tables

**TABLE I:** Comparison of Kinematic Factors Between the Early- (S1 and S2) and Late- (S9 and S10) Training Stages

Variables	Early-stage	Late-stage	*F*	*t*	δ

**AP COMx /BL**	**0.31±0.12**	**0.35±0.09**	**2.77** *	**−2.61** *	**0.41**
**ML COMx /BW**	0.41±0.06	0.42±0.06	0.95	0.42	0.08
**AP COMv (m/s**)	0.25±0.58	0.21±0.64	0.73	0.63	0.15
**ML COMv (m/s)**	0.21±0.1	0.22±0.08	0.36	0.78	0.04
**Step length (m)**	**0.17±0.08**	**0.20±0.07**	**2.73** *	**2.41** *	**0.43**
**Step width (m)**	0.21±0.06	0.21±0.06	2.1	0.11	0.06
**Initiation time (s)**	**0.25±0.08**	**0.22±0.05**	**4.04** *	**2.91** *	**0.49**
**Execution time (s)**	0.17±0.06	0.18±0.04	0.43	−0.79	0.28
**Trunk angle (°)**	99.8±6	98.7±5	0.92	0.36	0.19
**Arm flexion (°)**	24.2±5.6	23±13.2	0.37	0.63	0.07

Note: All spatial variables are at recovery foot touchdown.

Abbreviations: COMx denotes center of mass position, COMv denotes center of mass velocity, BL denotes BOS length, BW denotes BOS width.

* denotes p<0.05, and the values with significant changes between stages were bolded.

**TABLE II:** The Number of Participants Recruited Synergies with Different Dimensionality (from 3 to 6) in Early- and Late-Training Stages

Synergy Dimensionality (D)	D=3	D=4	D=5	D=6	Total

**Early-training stage**	8	9	9	0	26
**Late-training stage**	7	13	4	2	26

**TABLE III:** Comparison of Area Under Activation Curve and Peak Amplitude of Activation for the Synergy Modes Between Early and Late-Training Stages

Synergies	Early-stage	Late-stage	*p* value	Early-stage	Late-stage	*p* value
	Area under activation curve			Peak amplitude of activation		
**W**_TA_	**11.2±3.5**	**9.1±4.2**	**0.046**	0.6±0.13	0.61±0.28	>0.05
**W**_S_VLAT_	9.3±3.7	8.5±3.7	>0.05	0.59±0.16	0.57±0.21	>0.05
**W**_R_GAS_	7.8±2.4	8.9±2.4	>0.05	0.53±0.17	0.54±0.29	>0.05
**W**_R_VLAT_	9.5±3.4	7.7±3.4	>0.05	**0.58±0.17**	**0.44±0.18**	**0.01**
**W**_S_GAS_	6.4±2.5	7.3±4.7	>0.05	0.41 ±0.18	0.44±0.29	>0.05
	**Time of activation onset (%)**			**Time of peak activation (%)**		
**W**_TA_	36.6±8	35.5±9.5	>0.05	46.6±8.9	44±12	>0.05
**W**_S_VLAT_	41.1±12.4	41.3±9.5	>0.05	48.9±10.2	48.3±11.2	>0.05
**W**_R_GAS_	**44.9±9.3**	**35.2±11.6**	**0.001**	58.3±10.1	53.1±18.4	>0.05
**W**_R_VLAT_	44.5±15.8	48±14.5	>0.05	63.5±17.3	67.3±15	>0.05
**W**_S_GAS_	30.3±18.3	29.6±17.4	>0.05	45.4±24.4	47.7±17.6	>0.05

Abbreviations: W_TA_: Tibialis anterior dominant, W_S_VLAT_: Slipping side Vastus Lateralis dominant, W_R_GAS_: Recovery side Gastrocnemius dominant, W_R_VLAT_: Recovery side Vastus Lateralis dominant, W_S_GAS_: Slipping side Gastrocnemius dominant.
